# Improving Gander Reproductive Efficacy in the Context of Globally Sustainable Goose Production

**DOI:** 10.3390/ani12010044

**Published:** 2021-12-27

**Authors:** Muhammad Faheem Akhtar, Muhammad Shafiq, Ilyas Ali

**Affiliations:** 1 Jiangsu Province Key Laboratory for Molecular and Medical Biotechnology, College of Life Science, Nanjing Normal University, Nanjing 210023, China; 2 Research Institute of Donkey High-Efficiency Breeding and Ecological Feeding, College of Agronomy, Liaocheng University, Liaocheng 252000, China; 3 Department of Cell Biology and Genetics, Shantou University Medical College, Shantou 515063, China; drshafiqnjau@yahoo.com; 4 College of Animal Science and Technology, Nanjing Agricultural University, Nanjing 210095, China; ilyasnjau@gmail.com

**Keywords:** goose, gander re(production), improved reproductive efficiency

## Abstract

**Simple Summary:**

High economic gains from commercial poultry breeder stocks cannot be achieved by ignoring the importance of highly efficient male breeders. Male stock provides the basis for progeny and also ensures optimum fertility and hatchability. The present review is written to explore the application of various techniques that can assist in improving the reproductive efficiency of goose ganders, which exhibit poor reproductive performance. Recommended techniques for improving gander reproduction are the use of an artificial photoperiod, nutritional supplementation, monochromatic light sources, artificial insemination and semen cryopreservation, as well as immunization against the leptin hormone, anti-Müllerian hormone, and glycoprotein inhibin.

**Abstract:**

The goose is a popular poultry species, and in the past two decades the goose industry has become highly profitable across the globe. Ganders low reproductive performance remains a barrier to achieving high fertility and hatchability in subsequent flocks. To address the global demand for cheaper animal protein, various methodologies for improving avian (re)production should be explored. A large amount of literature is available on reproduction traits and techniques for commercial chicken breeder flocks, while research on improved reproduction in ganders has been carried out to a lesser extent. The present review aims to provide a comprehensive literature overview focusing on recent advancements/techniques used in improving gander reproductive efficacy in the context of ensuring a globally sustainable goose industry.

## 1. Introduction

Sustainable goose production cannot be achieved without focusing on all production aspects, i.e., nutrition, management, biosecurity, and reproduction. Management and feeding can directly impact reproduction rate, fertility, hatchability, and the number of eggs and goslings produced [1]. In 2019, the estimated total number of commercial poultry birds in the world was 27.9 billion, including 3.62 million geese and guinea fowl [2].

**Figure 1:**
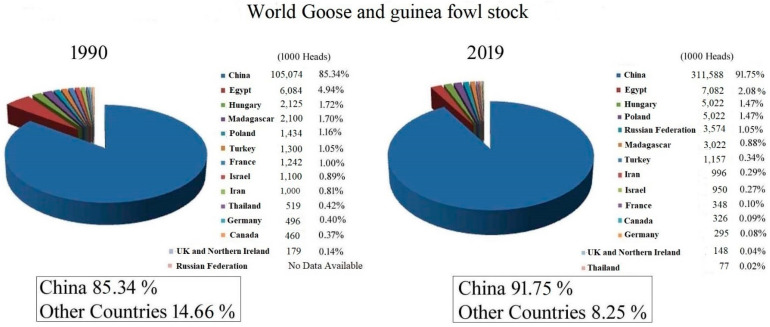
World goose and guinea fowl population in 1990 and 2019 according to FAOSTAT [2].

In 2021, the human population increased to 7.9 billion [3] and is expected to grow to 9.3 billion in 2050 [4]. This situation requires exploration of ways to provide better quality, more hygienic, and cheaper protein sources from plants and animals. During the past three decades, the global waterfowl industry has been remodeled [5]. Figure 1 shows the commercial geese and guinea fowl stock statistics three decades ago, in 1990, in comparison with the 2019 data. According to FAOSTAT [2], the world leaders in geese production are China, Egypt, Hungary, Poland, the Russian Federation, and Madagascar.

Geese are a popular source of eggs, meat, fat liver, goose fat, down, and feathers in numerous parts of the world [6,7,8,9,10]. Foie gras is a special food product obtained from duck and goose liver by gavage (force feeding) and is especially popular in French cuisine and countries such as Hungary, Poland, Israel, the USA, and Canada [9,10]. For more than 2000 years, goose feathers have been used for duvets and pillows [10]. Goose feathers and down originating from China, Hungary, Poland, and Canada are popular for filling mattresses, beddings, blankets, comforters, and furniture, having a lifespan of 50 years [9,10]. It is generally acknowledged that goose meat satisfies the human body’s need for many nutrients [11]. However, compared to other commercial poultry species (broilers, layers, quail, and turkeys), low performance overall and, specifically, the poor reproductive efficiency of male breeder stock remain barriers to achieving a holistic production approach [12].

Ancestors of present goose breeds are wild geese; the majority of goose breeds in Europe belong to the graylag goose (*Anser anser*), and in Asia and Africa, they originated from the swan goose (*A. cygnoides*) [13,14]. Based on genetic, phenetic, phylogenetic, and historical analyses, it was hypothesized that there are six centers of goose domestication, breed formation, and dispersion: namely West-European, Chinese, Euro-Asiatic, Egyptian, North-American, and Australian [15]. As a result of domestication, the phenotypic traits of geese have been altered, and their production performance improved [16].

Health, hygiene, and good management of breeder stock is essential for best performance in the goose production business. Males should make up 50% of the flock to achieve the maximum number of fertile hatching eggs [17]. Good reproductive characteristics, i.e., hatchability and fertility, are extremely important when determining reproduction in ganders [5]. Fertility and hatchability are major components in improving the reproductive efficiency of breeder stock [16] and fertility in males. Nutrition, housing system, bird health, genetics, sexual behavior, female age, sex ratio, temperature, and light can affect fertility [16].

To sustainably optimize goose production, there is dire need to explore the various reasons for lower reproductive efficiency in ganders and recent solutions. The present review summarizes the current status of the world’s goose industry in terms of reproduction and various aspects of poor reproductive efficiency along with recently developed methods to upgrade gander stock reproduction.

## 2. Why Do Ganders Have Poor Reproductive Performance?

The following are some reasons for the poor reproductive performance of ganders.

### 2.1. Seasonality

Due to the strong seasonality of egg laying, the goose industry has remained underutilized for the past two decades. Goose breeds in China (the highest producer of geese globally, accounting for 95% of world goose production) are seasonal breeders, and may differ in breeding seasonality depending on their native habitat or locations [8]. Seasonal breeder birds exhibit three states of testes activity, i.e., matured testes, matured and active testes, and matured but resting testes [18].

The progeny of all goose breeds are either long day breeders or short day breeders depending on the latitude. Depending on seasonality, goose breeds are divided into three types [19]:

1. Long day breeders: inhabitants of the higher latitude (40–45° N) temperate zone. They breed during the longer days in spring and early summer.
2. Inhabitants of the mid-latitude (30–40° N) temperate regions. Their breeding season starts in autumn and ends in the following spring–early summer.
3. Short day breeders: inhabitants of subtropical areas (22–25° N). Their breeding season starts from late summer to following spring.

In northern temperate zones, the majority of birds (including geese) have evolved altered endocrinological mechanisms (photoperiodism and photorefractoriness) that coincide with their breeding stage, characterized by periods of abundant food supply and supportive climatic conditions [18], particularly regarding photoperiod [19]. The purpose of this dynamic self-initiated alteration is to attain maximum reproductive efficiency. In short day breeder geese, spermatogenesis is depressed rather than considerably inhibited. In long day breeders, the testis and epididymis undergo marked structural changes to the extent that the testes lose shape and only Sertoli cells and spermatogonia remain in the seminiferous tubules.

Due to strong seasonality in breeding, ganders have lower testosterone (T) concentrations and semen quality specifically in the nonbreeding seasons [20]. Ganders decline in fertility after two months of high reproductive efficiency with concomitant marked changes [21] in sexual behavior, endocrine parameters, and semen quality [21,22]. In peak breeding and nonbreeding seasons, marked changes and steroidogenic activity are observed in ganders [23]. Annual changes in the photoperiod alter the pituitary secretions of gonadotrophins during breeding seasons [19]. In birds, the anterior pituitary determines testes endocrinology through the luteinizing hormone (LH) and follicle stimulating hormone (FSH) via hypothalamic gonadotrophin-releasing hormone (GnRH) pulsatile secretion [24,25,26]. It is known that geese have a lower fertility and hatchability rate (53.8–84.72% and 61–63%, respectively) [16] compared to chickens (89–94% and 90–92%) [27] and ducks (75.9% and 57.68%, respectively) [28], which are further decreased during the breeding season.

### 2.2. Poor Semen Quality

Geese have the lowest reproductive efficiency of all poultry species, in addition to relatively poor semen quality and fertility [29]. One-year-old ganders have a large semen volume and a lower proportion of morphologically normal live spermatozoa compared to 2-year-old ganders [30]. Irrespective of age, the percentage of total live sperm was 91–95% but was 27–41% for morphologically normal sperm, with high percentages of defective sperm (bent neck, macrocephalic) [31]. Gumulka and Rozenboim [22], El-Hanoun et al. [32], and Kowalczyk and Lukaszewicz [33] observed lower sperm concentration and live spermatozoa count in the second half of the breeding season in ganders. The decrease in fertility in the second half of the breeding season may be associated with depressed semen quality and lower T concentrations [34]. From this work, it can be concluded that semen quality declines faster for ganders than cocks.

Prolactin (PRL) seems to mediate lower T and LH concentrations [26]. In Magang ganders, T concentration followed LH concentration, while the seasonal pattern of PRL was opposite to LH and T [29]. The transition from a long to a short photoperiod stimulated testicular development and semen production, while from short to long photoperiod decreased semen and testicular size and arrested spermatogenesis in ganders. The LH receptor is positively correlated with T concentrations [35]. Seasonally, a high concentration of PRL is a negative regulator of LH, T [22], and semen quality in domestic ganders [36]. In various goose breeds, sperm quality had been improved by using artificial insemination (AI) and natural breeding [37]. In ganders, such factors as feeding, semen collection method, growing system, semen collection frequency, and breed traits affect sperm quality [38].

### 2.3. Age of Maturity

The traits for male breeder stock to optimize commercial farming are body weight (BW), feed efficiency, muscle growth, livability, age of maturity, and fertility. Compared to other commercial poultry species such as broilers, layers, ducks, pigeons and turkeys, geese take a longer time to mature. It was noted that 1-year-old geese mature at the age of 279.7 ± 1.8 days [21]. The age of sexual maturity for Yangzhou ganders is 227 days [5].

Breeding companies are focused on selecting superior performance in all traits of economic importance [39]. The feed conversion ratio is the most important trait to determine how much daily weight is gained after consuming how much feed. Poultry feed is the major cost of poultry production, i.e., 70% of poultry production. Hence, from the economic aspect, normally farmers prefer chickens because their return on investment is quicker, but there is a higher likelihood of greater investment in countries with a high consumer demand for goose meat and eggs. Pullets mature at 21 weeks of age, and broilers reach 2–2.2 kg at 38–42 days of age [40], while the majority of small, medium, and large sized goose breeds in the world mature after 200 days of age.

Commercial breeder stocks in poultry (including geese) are meant to exhibit efficient growth, increased muscle yield, and improved genetic potential [41,42,43,44]. In comparison with other commercial poultry male breeder stocks, it takes longer to develop male progeny of high genetic potential and earlier maturity in ganders despite the greater efforts and use of a wider variety of techniques.

## 3. Methods to Improve Reproductive Efficiency of Ganders

The following are various methods adopted by researchers to improve the reproductive efficiency of ganders.

### 3.1. Artificial Photoperiod and Monochromatic Light Sources

As discussed previously, to maximize genetic potential, reproductive activity is synchronized with changing seasons for the majority of geese, i.e., when food is plentiful, and the conditions are most optimal for the growth of their goslings. In wild goose species, this synchronization is beneficial; however, in commercial goose farming it remains a barrier to maximizing economic gains. Sun et al. [45] successfully introduced artificial lighting programs, also called out of season geese laying, that improved geese reproductive performance and resulted in profits four to six times higher than using the natural photoperiod [35].

In avian species, the anterior pituitary secretes gonadotropins LH and FSH that control the testes, while Leydig cells steroidogenesis is initiated and maintained by LH [46,47]. LH secretion is positively correlated with T concentrations and negatively correlated with PRL secretions [35,36]. High levels of PRL regress reproductive activities in birds [48,49]. Hence, altering the photoperiod of ganders, i.e., to the short photoperiod for Magang ganders, caused waning of PRL secretion and activation of GnRH, leading to elevated LH [35] that stimulated T concentrations. T is further utilized by Sertoli cells for nourishment of germ cells such as spermatogonia and spermatocytes for smooth progression of spermatogenesis [5]. In ganders, altering the photoperiod can help optimize reproductive performance. Gumulka and Rozenboim [22] concluded that T concentration is a poor indicator of semen production in Zatorska ganders.

Artificial illumination promotes poultry reproduction and growth [50,51]. Varying light wavelengths exerts different stimulatory effects on retina photoreceptors, which affects gonadal development and efficiency [52,53,54]. Monochromatic white, red, and blue light were applied to 3-year-old Roman ganders, and only white light enhanced semen quality, motility, viability, and percentage of normal spermatozoa [55]. Egg production and activation of the reproductive system was achieved after white and red light-emitting diode illumination with a long photoperiod of 11 h in Yangzhou geese [56].

Various light colors directly affect reproductive organ development, sexual maturity, muscular development, and behavior [57,58,59]. Green light accelerated embryo development and earlier hatching in chickens [60,61]. Green light stimulated lowered hatching time in four layer breeds during embryogenesis [62]. Reproductive performance of White King pigeons was improved by blue (480 nm), red (660 nm), and green (540 nm) light stimulation for three months [63].

Poultry production and behavior are directly affected by different wavelengths of light, though color illumination has been reported in many reports [64]. Broilers gained more BW under blue and green light compared to red light [65]. Improved fertility and chick production was observed in broiler breeders using green light [66]. Green light improved egg quality in laying hens [67]. Growth, T concentrations, and skeletal myofiber growth was observed in broilers using green and blue light illumination [51]. These results suggest that monochromatic light has a tendency to increase T, egg quality, fertility, semen quality, BW gain, and hatchability in commercial poultry birds.

### 3.2. Nutritional Supplementation

In 3-year-old White Koluda ganders, manual semen collection and semen quality were improved after dietary supplementation of commercial feed with selenium (0.3 mg/kg) and vitamin E (100 mg/kg) [68]. In breeder geese, dietary supplementation with micronutrients (essential amino acids, vitamins, and trace elements) dissolved in drinking water at a daily dose of 50 g/500 kg weight, 10 days before the onset of the laying period, followed by a 10 day interval without supplementation and then supplementation for 10 days until the end of laying period, improved egg production and laying intensity, but seldom improved egg fertility and hatching rates of goslings [69]. In other commercial avian breeder stock, addition of feed additives improved reproduction as well as semen quality.

Lycopene supplementation at 300, 600, and 900 mg/kg elevated semen biochemical traits and improved reproductive performance [70]. In another study, 5 g/L lycopene in drinking water improved fertility, sperm volume, and viability in roosters [71]. Moreover, 0.05 mg/mL lycopene in drinking water reduced oxidative damage in semen during cryopreservation in addition to elevating sperm viability [72].

Supplementation with the oxidative compound L-carnitine (LC) at doses of 50 and 150 mg/kg of BW for 12 weeks showed significant elevated seminal antioxidant enzymes and may ultimately improve semen quality in aging cocks [73]. Dietary supplementation with 125 mg/kg LC increased sperm concentration in White Leghorn roosters [74] and viability in motility quail [75]. Administration of 150 mg LC/kg in the diet elevated sperm concentration, semen volume, and lowered concentration of defective sperm in male ducks [76]. LC supplementation improved sperm membrane functioning and increased mitochondrial and testicular activity [77,78,79], with elevated T, spermatogenesis, and sperm functioning [73,78].

Supplementation of vitamin E and selenium yeast resulted in improvements such as increased hatchability, post hatch growth during the first 7 days, fertility, and embryo weights in guinea fowl [80]. Manganese (Mn) supplementation of 10 mg/kg of feed significantly increased sperm mass and lowered the frequency of abnormal sperm in cocks, while in dual purpose cross bred hens, Mn in the control diet (16 mg/kg from raw materials and 20 mg/from organic or inorganic source) can profoundly improve reproductive efficiency, egg production, quality, and economic gains [81]. D-aspartic acid is present at adequate levels in the testes of mature ducks during reproductive seasonality and its addition beneficially increased T secretion in vitro [82], while in aged roosters, reproductive parameters (semen quality, sperm concentration, penetration, motility, and membrane integrity) were increased at 200 mg/kg BW [83]. Adding guanidinoacetic acid (GAA) at 1200 mg/kg for four weeks upgraded quail fertility [84], while the same GAA dosage elevated fertility in roosters [85]. Similarly, dietary supplementation of ginger increased antioxidant capacity in chickens and laying hens [86,87]. In male broiler breeders, dietary supplementation of 50 mg chrysin improved fertility and semen quality [88,89].

### 3.3. Inhibin Immunization

Inhibin (INH) is a heterodimeric glycoprotein (31–34 KDa) found as two isotopes i.e., INH A (α-βA) and INH B (α-βB), that share a common α subunit, differ in the β subunit, and play important roles in the hypothalamus pituitary gonadal (HPG) axis [90,91,92]. INH is a negative feedback regulator of FSH [93]. INH is secreted by Sertoli cells in male testes and granulosa cells of ovarian follicles in females [94]. In males, FSH directly acts on Sertoli cells that nourish germ cells, ultimately negatively effecting reproductive efficiency.

INH immunization is an effective tool for improving reproductive performance in various commercial animals and bird species. In Yangzhou ganders, immunization against the INH α-subunit improved testicular weight and Sertoli cell development but lowered T concentration [95]. Plasma T concentrations are not standard for semen quality in goose flock management [22]. In Japanese quail, INH immunization expedited puberty and elevated hen-day egg production [96]. In Partridge Shank hen, the INH vaccine enhanced the production of antibodies against INH, follicular development, and egg production. In hens from juvenile state to sexual maturity, INH immunization upgraded rate of ovulation and follicular development without increasing FSH [97]. Immunization against the INH α subunit promoted testicular development in developing cockerels [98] without effecting FSH. However, INH immunization did not significantly enhance egg production in turkey hens [99]. α-INH immunization improved daily sperm production in rams [100].

### 3.4. Leptin Immunization

Leptin (LEP) is a peptide hormone secreted from white adipocytes that plays a vital role in reproduction, nutrition, and energy reserves [101,102,103]. LEP plays an important role in regulation of reproduction and fertility via the HPG axis [104,105]. To explore the function of LEP protein and to improve reproductive efficiency in animals, LEP immunization was practiced in Yangzhou ganders and resulted in lowered T, sperm counts, and testes weight [106].

Immunization against the LEP receptor downregulated egg production and follicular development and lowered fat deposition in growing chickens [107,108]. Higher body fat is directly proportional to lower sperm concentration, sperm motility, and T in males [109,110]. LEP plays an important role in fertility and pubertal development [111]. LEP immunization lowered sperm count and motility in adult rats [112]. LEP immunization at 3 mg/kg lowered sperm concentration and motility, and an increased dose resulted in damaged testes [113].

### 3.5. Artificial Insemination and Semen Cryopreservation

In AI, semen is transferred into a female’s vagina. The purpose of AI is to produce fertilized eggs between inseminations [114]. AI is extensively used in turkeys [114]. AI improved percentage hatchability and fertility compared to natural mating in guinea fowl [115]. A greater number of fertilized eggs was obtained in broiler breeder hens after application of AI [116]. Fertility rates after AI were elevated up to 80% in quail, being equivalent to natural mating results [117]. AI in large parrots proved a milestone in a species conservation program [118]. AI in poultry species results in efficient use of best sires at pedigree farms, i.e., one male and ten to twelve females compared to the conventional mating ratio of one male and three to four females [38]. For high egg production in ostrich, AI can replace the male presence for breeding [119].

Semen cryopreservation is an important method for storing reproductive cells and ensuring the genetic diversity of birds [120,121]. The genetic potential of wild and domestic birds can be preserved by semen cryopreservation [29,120,122]. A satisfactory fertility level was obtained with frozen–thawed semen in various poultry species [29,123,124]. The semen collection procedure is an important factor for gander quality semen [31,125,126]. Semen cryopreservation significantly affected semen quality parameters in 1-year-old ganders compared to older ganders [29]. The semen cryopreservation method worked effectively for White Koluda, graylag [127], and Large White geese [128]. Frozen–thawed semen of Chinese ganders can improve the fertility of White Roman geese at the end of the production season [129].

Due to semen compositional variations among mammalian and avian species, semen conservation techniques can be complicated and require further advancements as conditions vary among avian species. In addition, keeping avian semen fertile in the female genital tract after AI is another challenge as compared to mammalian species [122]. AI and semen cryopreservation are proven techniques for farm animal breeding. Improving fertility and hatchability requires technical assistance for semen collection, storage, and further application in breeder stock. Improper AI results in poor results [130]. Thawed semen alone cannot achieve improved fertility, and hatchability of eggs can also be higher [16]. The introduction of experienced ganders is recommended as they produce higher quality semen and copulations [31,38,131,132]. Before the onset of the reproductive season, male breeders should be preselected to ensure a sufficient amount of semen for female insemination [133].

### 3.6. Anti-Müllerian Hormone

The anti-Müllerian hormone (AMH) plays a vital role in Müllerian duct regression in the male fetus [134]. Serum AMH is a marker of Sertoli cell development [135,136]. The Sertoli cell is the central regulator of testis development [137]. Sertoli cells of fetal mammalian species secrete AMH [138]. Upregulation of AMH gene mRNA expression indicates Sertoli cell development [95]. AMH causes regression of the uterus, upper vagina, and fallopian tubes in female mammals [139]. In the human ovary, AMH is initially expressed in the granulosa cells of primary follicles, peaks in antral and small antral stages of follicular development, and subsequently declines [140].

Compared to mammals, the literature on AMH expression in avian species is scarce [141,142,143]. In broody Zhedong White geese, active immunization against the AMH protein elevated development of small yellow and preovulatory follicles that resulted in increased clutch size [144]. However, AMH active immunization is not yet well reported in ganders and needs to be explored in terms of gonadal function.

## 4. Conclusions

Low reproductive performance is a known problem of ganders. To optimize performance, a holistic approach should be adopted for improving breeder geese stock performance, including consideration of animal husbandry practices, disease prevention, staff training, crossbreeding, nutritional modulation, and biosecurity. Crossbreeding is also a proven method for developing more commercially productive strains, e.g., Yangzhou geese. Poultry production requires more attention from geese researchers and practitioners on all aspects of production, including improvement of gander reproduction. The development of breeds and keeping geese parent stocks are also economically prohibitive for research purposes and requires improved collaboration between academia and industry.

## Data Availability

Data is contained in cited references.
